# Radiolabeled 6-(2, 3-Dichlorophenyl)-N4-methylpyrimidine-2, 4-diamine (TH287): A Potential Radiotracer for Measuring and Imaging MTH1

**DOI:** 10.3390/ijms21228860

**Published:** 2020-11-23

**Authors:** Huaping Chen, Sadia Afrin, Yingqiu Guo, Wenhua Chu, Tammie L.S. Benzinger, Buck E. Rogers, Joel R. Garbow, Joel S. Perlmutter, Dong Zhou, Jinbin Xu

**Affiliations:** 1 Department of Radiology, Washington University School of Medicine, St. Louis, MO 63110, USA; chenhuaping@wustl.edu (H.C.); sadia@wustl.edu (S.A.); yingqiu.guo@duke.edu (Y.G.); chuw@wustl.edu (W.C.); benzingert@wustl.edu (T.L.S.B.); garbow@wustl.edu (J.R.G.); perlmutterjoel@wustl.edu (J.S.P.); 2 Department of Radiation Oncology, Washington University School of Medicine, St. Louis, MO 63110, USA; b.rogers@wustl.edu; 3 Department of Neurology, Washington University School of Medicine, St. Louis, MO 63110, USA

**Keywords:** MTH1, radiotracer, TH287, binding assay, PET

## Abstract

MTH1 (MutT homolog 1) or NUDT1 (Nudix Hydrolase 1), also known as oxidized purine nucleoside triphosphatase, has potential as a biomarker for monitoring cancer progression and quantifying target engagement for relevant therapies. In this study, we validate one MTH1 inhibitor TH287 as a PET MTH1 radiotracer. TH287 was radiolabeled with tritium and the binding of [^3^H]TH287 to MTH1 was evaluated in live glioblastoma cells (U251MG) through saturation and competitive binding assays, together with in vitro enzymatic assays. Furthermore, TH287 was radiolabeled with carbon-11 for in vivo microPET studies. Saturation binding assays show that [^3^H]TH287 has a dissociation constant (*K*_d_) of 1.97 ± 0.18 nM, *B*_max_ of 2676 ± 122 fmol/mg protein for U251MG cells, and *n*_H_ of 0.98 ± 0.02. Competitive binding assays show that TH287 (*K*_i_: 3.04 ± 0.14 nM) has a higher affinity for MTH1 in U251MG cells compared to another well studied MTH1 inhibitor: (S)-crizotinib (*K*_i_: 153.90 ± 20.48 nM). In vitro enzymatic assays show that TH287 has an *IC*_50_ of 2.2 nM in inhibiting MTH1 hydrolase activity and a *K*_i_ of 1.3 nM from kinetics assays, these results are consistent with our radioligand binding assays. Furthermore, MicroPET imaging shows that [^11^C]TH287 gets into the brain with rapid clearance from the brain, kidney, and heart. The results presented here indicate that radiolabeled TH287 has favorable properties to be a useful tool for measuring MTH1 in vitro and for further evaluation for in vivo PET imaging MTH1 of brain tumors and other central nervous system disorders.

## 1. Introduction

Improved cancer diagnosis and cancer-specific therapeutics are needed to reduce cancer mortality rates. Positron emission tomography (PET) can measure disease-based changes in cell function at the molecular level. PET combined with computed tomography (CT) or magnetic resonance imaging (MRI) provides a powerful hybrid of anatomical and functional assessments of cancer and other diseases.

Dysfunctional redox regulation and increased reactive oxygen species (ROS) cause oxidative damage to nuclear and mitochondria DNA that contributes to the pathogenesis and progression of cancer and other diseases [1]. Thus, in vivo measures of oxidative stress or related DNA damage repair through PET could provide a critical metric of disease progression and a target for drug intervention. Furthermore, PET results on oxidative DNA damage can serve as a prognostic indicator for radiotherapy in cancer patients [2,3] since ionizing radiation can directly interact with water in the biological systems to produce ROS and subsequent oxidative DNA damage, in addition, to directly cause DNA breaks. Aerobic glycolysis is also a marker of tumor growth and aggressiveness [4]. Therefore, a reliable biomarker targeting DNA damage repair could be valuable for monitoring cancer progression and evaluating response to chemo- or radio-therapeutics, as well as pseudo-progression and radiation necrosis, which are difficult to separate clinically using currently available MRI and PET imaging approaches [5].

Cancer cells require high ROS concentrations due to their high proliferation rate. In mitochondria, where ~90% of total oxygen consumption occurs, about 1–4% consumed oxygen molecules are partially reduced by electrons to generate ROS including superoxide, hydrogen peroxide, and hydroxyl radicals [6]. ROS causes cellular dysfunctions such as cell death and mutagenesis by damaging lipids, proteins, and nucleic acids in the living cells [7]. Guanine is the most susceptible nucleobase to be oxidized by hydroxyl radicals, a reaction that results in the formation of 8-oxo-2’-deoxyguanosine (8-oxo-dG) or 8-oxo-2’-deoxyguanosine-5’-triphosphate (8-oxo-dGTP) [8]. The concentration of 8-oxo-dG within a cell reflects the degree of oxidative stress [9].

Misincorporation of oxidized nucleoside triphosphates into DNA/RNA during replication and transcription can cause mutations that may lead to carcinogenesis, senescence, apoptosis, or neurodegeneration [10,11]. MTH1, or NUDT1, an oxidized purine nucleoside triphosphatase, hydrolyzes oxidized purine nucleoside triphosphates, such as 8-oxo-dGTP, 8-oxo-dATP, 2-hydroxy-dATP, and 2-hydroxy rATP, to monophosphates. These molecules suppress the accumulation of oxidative damage in nucleic acids and prevent the misincorporation of the oxidized nucleoside triphosphates into DNA/RNA [11,12,13,14,15,16]. MTH1 is upregulated in a wide variety of cancers, including brain tumors [17,18], lung cancer [19,20], breast cancer [21,22], liver cancer [23], renal cancer [24], colorectal cancer [25], esophageal cancer [26], gastric cancer [27], and multiple myeloma [28]. Furthermore, as tumors progress to advanced stages, MTH1 expression upregulates. MTH1 levels also correlate with the overall survival of HCC (Hepatocellular carcinoma) patients [23].

**Figure 1:**
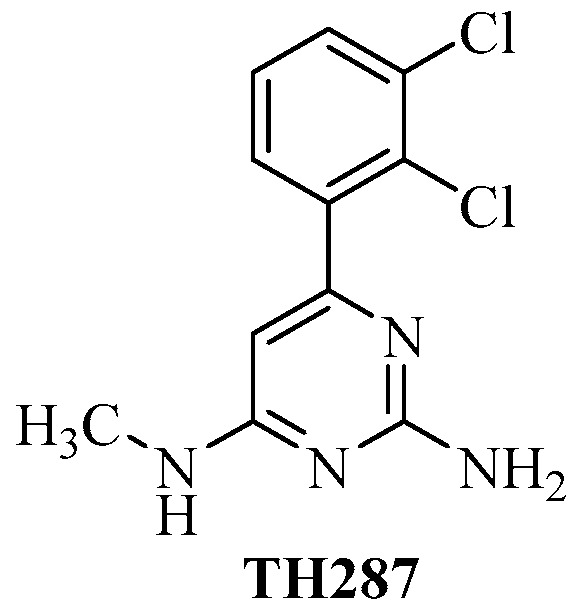
Chemical structure of TH287.

Thus, monitoring MTH1 status in patients through in vivo imaging would facilitate the design of therapeutic strategies that target oxidative DNA damage involved diseases such as cancer. TH287 (Figure 1) is among the known MTH1 inhibitors and is a potent first-in-class molecule that inhibits MTH1 specifically in in vitro enzymatic assays [29]. However, whether TH287 has a high affinity to MTH1 in live cells remains unknown.

In the present study, the affinity of TH287 to MTH1 in live glioblastoma U251MG cells and in vivo was investigated using its radiolabeled form. TH287 was radiolabeled with tritium and the binding of [^3^H]TH287 to MTH1 in glioblastoma cells (U251MG) was evaluated in vitro using saturation binding, competitive binding, and enzymatic assays. Furthermore, TH287 was radiolabeled with carbon-11 to study whether [^11^C]TH287 is a suitable radiotracer for PET. The uptake and distribution of [^11^C]TH287 in different organs of mice were investigated with microPET imaging. This is the first study that explores the feasibility of developing a radiolabeled MTH1 inhibitor for use as an in vitro binding assay or in vivo PET imaging radiotracer.

## 2. Results

### 2.1. Saturation binding assays in U251MG cells

**Figure 2:**
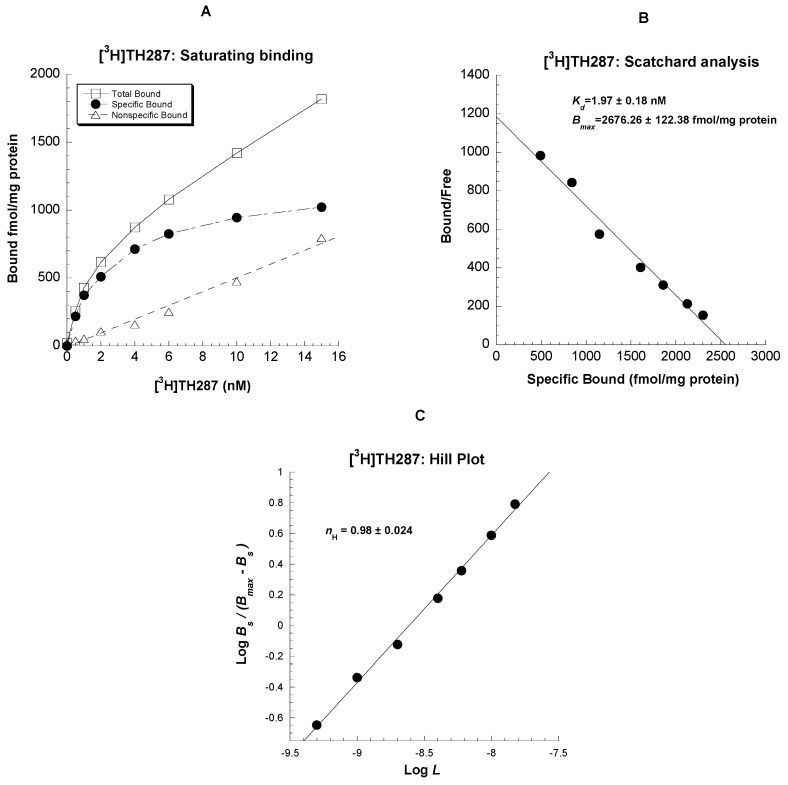
Scatchard analysis of [^3^H]TH287 binding to the MTH1 in glioblastoma cells (U251MG). (**A**): Representative saturation binding experiments that show the total bound, non-specific bound, and specific bound. (**B**): Representative Scatchard plots were used to determine *K_d_* and *B_max_* values. *K_d_* = 1.97 ± 0.8 nM, *B_max_* = 2676.26 ± 122.38 fmol/mg protein. (**C**): Representative Hill plot for determining the Hill coefficient (*n*_H_ values). *n*_H_ = 0.98 ± 0.024. n = 3, samples in triplicate.

To validate TH287 as a potential radiotracer for microPET imaging, its affinity to MTH1 was first characterized in live cells through saturation binding studies. The assays were carried out by incubating increasing concentrations of [^3^H]TH287 with U251MG cells. The saturation curve, Scatchard plot, and Hill plot are shown in Figure 2. [^3^H]TH287 has a *K*_d_ value of 1.97 ± 0.18 nM, a *B_max_* value of 2676.26 ± 122.38 fmol/mg protein, and a *n*_H_ value of 0.98 ± 0.024 for U251MG cells. These values suggest TH287 is within the pharmaceutic limit to be adopted as a radiotracer for microPET imaging.

### 2.2. Knockdown MTH1 Expression through siRNA Abolishes [^3^H]TH287 Binding to U251MG Cells

**Figure 3:**
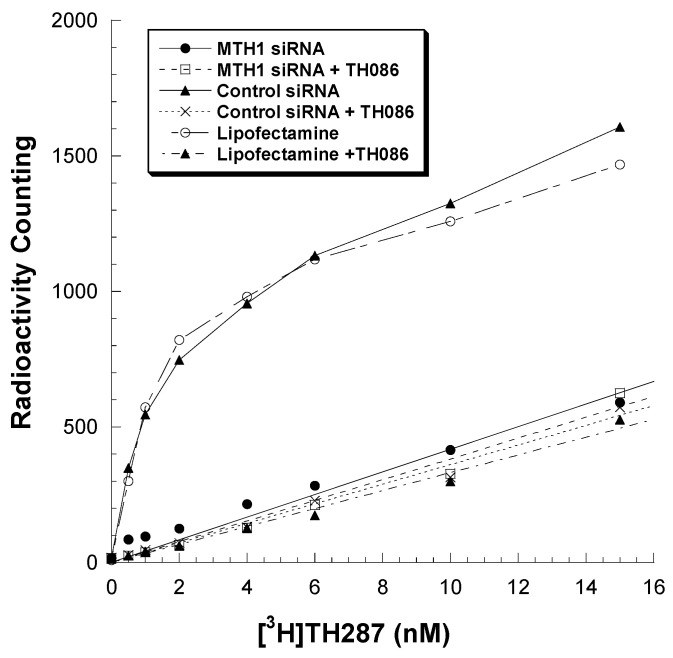
Saturation binding assay for [^3^H]TH287 to the MTH1 in MTH1 knockdown glioblastoma cells (U251MG). Representative saturation binding experiments which show the binding of [^3^H]TH287 in control groups and MTH1 knockdown groups. n = 3, samples in triplicate.

To exclude potential off-target effects caused by [^3^H]TH287 in the saturation binding assays, MTH1 was further knocked down through reverse transfection of MTH1 siRNA into U251MG cells. MTH1 knockdown completely abolished [^3^H]TH287 binding as shown by radioligand binding readings of MTH1 siRNA transfection groups similar to cells treated with 10 µM TH086, which is normally considered as non-specific binding, suggesting that [^3^H]TH287 binds specifically to MTH1 in U251MG cells instead of other targets (Figure 3).

### 2.3. Competitive Profile of Standard and TH287 in U251MG Cells

**Figure 4:**
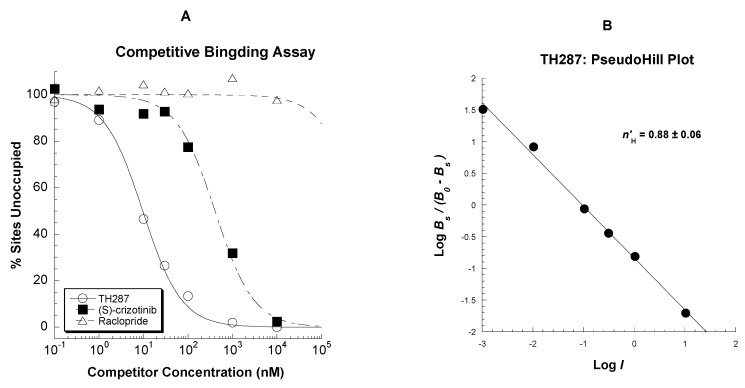

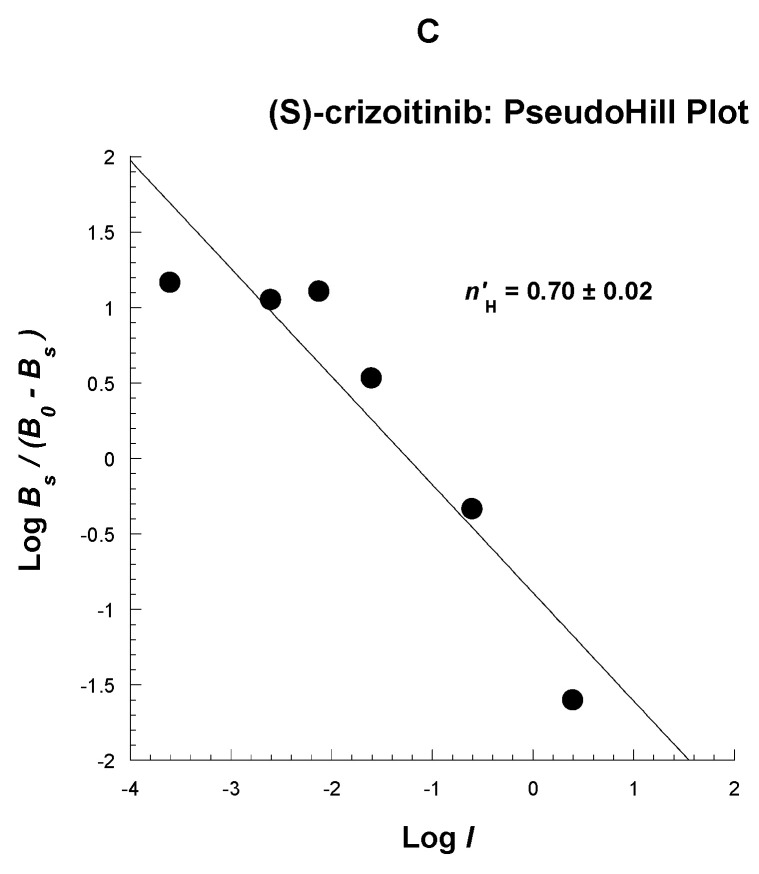
Competitive binding for inhibition of the [^3^H]TH287 binding to MTH1 in glioblastoma cells (U251MG) by TH287, (S)-crizotinib and a known MTH1 nonselective compound: Raclopride. (**A**): Representative competitive binding data *K*i for TH287: 3.04 ± 0.14 nM, (S)-crizotinib: 153.90 ± 20.48 nM, Raclopride: >10,000 nM. (**B**) (TH287) and (**C**) ((S)-crizotinib): Representative PseudoHill plots for determining the PseudoHill coefficient (*n’*_H_ values). TH287: *n’*_H_ = 0.88 ± 0.06; (S)-crizotinib: *n’*_H_ = 0.70 ± 0.02. n = 3, samples in triplicate.

To further validate the potential of TH287 as a radiotracer for MTH1, full dose range competition binding assays were performed between a well-studied MTH1 inhibitor, (S)-crizotinib, and [^3^H]TH287. Competitive binding assays show that TH287 has a high binding affinity, with a *K*_i_ of 3.04 ± 0.14 nM while (S)-crizotinib shows a *K*_i_ of 153.90 ± 20.48 nM, indicating that its affinity is much lower than TH287. No binding was detected for Raclopride—a nonselective compound (Figure 4). The values of the pseudo-Hill coefficient, *n’*_H_, are 0.88 and 0.70 for TH287 and (S)-crizotinib respectively.

### 2.4. Inhibition Profile of Standard and TH287 on Enzyme Activity of MTH1

**Figure 5:**
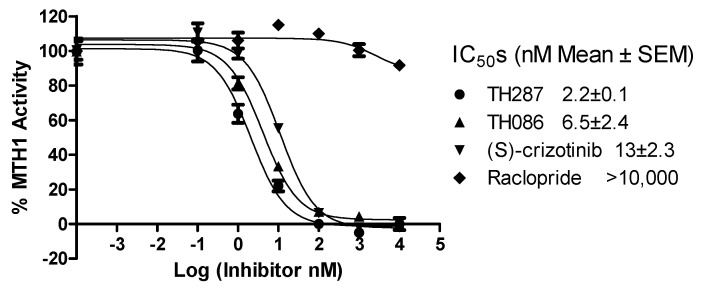
MTH1 inhibition assay shows that TH287, TH086, and (S)-crizotinib have high inhibition potency, with *IC*_50_s 2.2, 6.5, and 13 nM respectively. While a non-active compound Raclopride was used as a control compound; n = 2, samples in triplicate; substrate dGTP concentration: 100 uM, and recombinant MTH1 protein: 0.5 nM.

**Figure 6:**
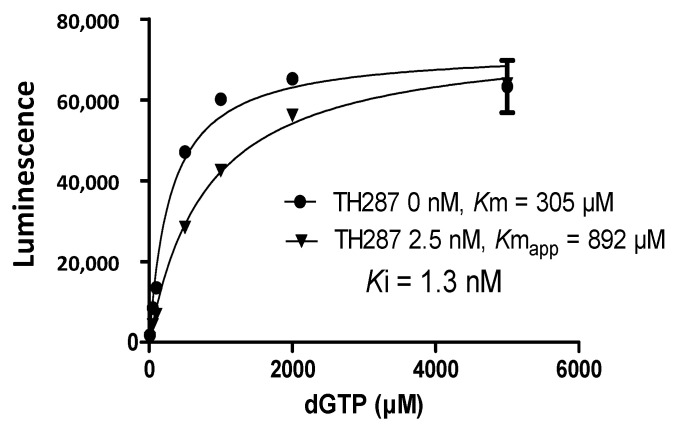
Representative competitive inhibition of MTH1 by TH287 (MTH1 0.5 nM, substrate dGTP ranges from 0 to 5000 uM) determined *K*i of TH287 to be 1.3 nM. *K*m: the Michaelis constant; *K*m_app_: apparent *K*m; *K*i: the inhibitory constant. n = 3, samples in triplicate.

To confirm the inhibition potency of TH287, a MTH1 enzymatic inhibition assay using a PPiLight^TM^ inorganic pyrophosphate bioluminescent assay (Lonza Walkersville Inc., MD, USA), was conducted to compare inhibition efficacy of TH287, TH086, and (S)-crizotinib (Figure 5). The MTH1 inhibition assay shows that TH287, TH086, and (S)-crizotinib have high inhibition potency with *IC*_50_s at 2.2, 6.5, and 13 nM respectively. While the non-selective compound, Raclopride shows no inhibition of MTH1 activity. Furthermore, competitive inhibition assays were performed and the *K*i of TH287 was determined to be ~1.3 nM (Figure 6). When TH287 is absent in the enzyme reaction, the Km for MTH1 is 305 µM. When 2.5 nM of TH287 is present in the reaction, the *Km*_app_ of MTH1 is 892 µM. This suggests that TH287 competes with the substrate to bind to MTH1.

### 2.5. TH287 Exhibits no Killing Effect on Cells

**Figure 7:**
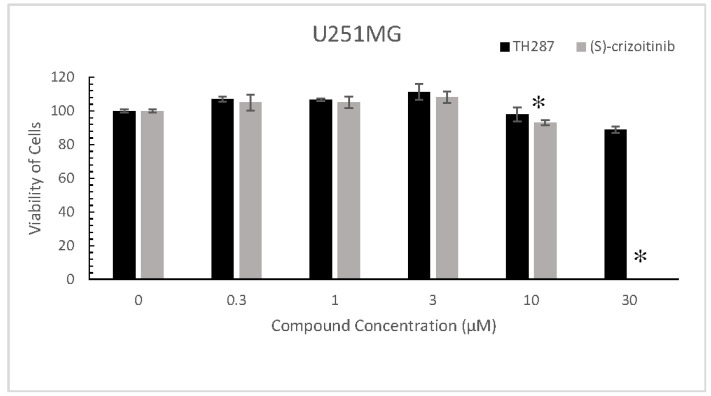
The effect of (S)-crizotinib and TH287 treatment on the viability of U251MG cells by MTT assay. U251MG cells were treated with increasing concentrations of (S)-crizotinib or TH287 (range: 0~30 µM) for 72 h, MTT assays were then performed. One Way ANOVA and Dunnett’s Multiple Comparison test were adopted for significance analysis. In assessing the significance of comparisons a *p*-value *p* < 0.05 was considered significant. *, *p* < 0.05. n = 3, samples in triplicate.

To test the toxicity of TH287 to cancer cells, U251MG cells were treated with TH287 ranging from 0.3~30 µM for 72 h. The same doses of (S)-crizotinib were also included in the experiments to compare with TH287. TH287 exhibits no cytotoxic effect on U251MG at the majority of the doses tested except slight proliferation inhibition at 30 µM (88.93%). While 30 µM (S)-crizotinib killed all the cells within 72 h of treatment (0.14%) and showed 92.95% cell viability with a 10 µM treatment for 72 h compared with the vehicle control (Figure 7).

### 2.6. MicroPET Imaging

**Figure 8:**
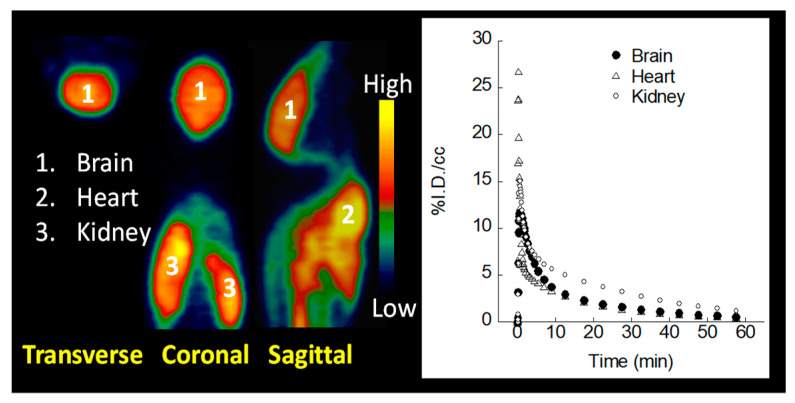
Representative summed (0–10 min) frames microPET images (left panel) from n = 4 male severe combined immunodeficient mice (SCID) mice (~25 g, 3 months old) show that [^11^C]TH287 had rapid brain uptake and fast clearance in a normal mouse; right panel shows [^11^C]TH287 time-activity curves in brain, heart, and kidney.

To validate the potential of TH287 as a radiotracer for PET imaging on tumor detection, [^11^C]TH287 was synthesized and microPET imaging was performed in normal mice. In the microPET imaging of normal mice, summed (0–10 min) frames of the microPET images (Figure 8 left panel) show that [^11^C]TH287 (~0.4 mCi) had high initial uptake and fast clearance of [^11^C]TH287 from the brain, heart, and kidney (Figure 8 right panel).

## 3. Discussion

In this study, we explored the feasibility of developing TH287 as a PET radiotracer for cancer by investigating the affinity of TH287 to MTH1 in live cells and its distribution and clearance in mice. TH287 exhibits a high affinity for MTH1 in the nanomolar range (*K*_i_: 3.04 ± 0.14 nM). Several existing MTH1 inhibitors besides TH287, including TH086 and (S)-crizotinib, were also tested in the study to validate TH287 as a specific MTH1 probe. TH287 has a higher affinity to MTH1 compared to all these two ligands. The cell viability studies showed that up to 30 µM TH287 exhibits minimal inhibition on U251MG cells while 30 µM (S)-crizotinib killed all the cells via an off-target other than MTH1. MicroPET imaging shows that TH287 penetrates the blood-brain barrier of mice with a fast clearance.

During this research, several potent MTH1 inhibitors have been synthesized and validated, including Tetrahydronaphthyridine 5 (*IC*_50_ = 0.043 nM) [30], BAY-707 (*IC*_50_ = 2.3 nM) [31], and other compounds [32]. Although these compounds exhibit a much higher affinity compared with existing inhibitors, none of them show potent cancer-killing efficacy, casting doubts on the potential of MTH1 to provide effective cancer treatment drugs. However, these ligands may still provide useful biomarkers for quantifying the treatment efficacy of current or new therapies.

Huber et al. showed that overexpression of MTH1 could rescue SW480 cells from being killed by treatment with a MTH1 inhibitor, SCH5134 but not with (S)-crizotinib [33]. This indicates that (S)-crizotinib could have other targets beyond MTH1. Here the cell viability data shows that up to 30 µM of TH287 treatment for 72 h exhibits minimal growth inhibition on U251MG cells, while the same dose of (S)-crizotinib kills all the cells. This suggests that (S)-crizotinib may also have different targets compared with TH287, although both molecules do target MTH1, which is consistent with Huber’s data. Kawamura et al. showed that TH287 and TH588 can inhibit in vitro beta-tubulin polymerization at concentrations higher than 30 µM [34]. However, Helleday et al. later showed that TH588 exhibits a different mode of action compared to the anti-microtubule agent. This casts doubts on the opinion that tubulin is an off-target of TH287 and TH588—especially considering the high doses of TH287 and TH588 used in Kawamura’s study [35]. TH1579 (Karonudib), an analog of TH588, can cause a toxic 8-oxodG lesion into DNA in an MTH1-dependent manner. While MTH1 inhibitors (compounds 15, 19, 24, and IACS-4759), which have no toxicity to tumor cells failed to incorporate 8-oxodG into DNA [35], suggesting the cancer cell killing effect of TH287 and TH588 is not necessarily caused by off-target effects. Further study by Helleday et al. showed that MTH1 can also bind to tubulin and promote mitotic progression to avoid oxidative DNA damage in cancer cells [36]. Those cytotoxic MTH1 inhibitors can inhibit both roles of MTH1 while newly developed non-cytotoxic inhibitors can only inhibit its enzymatic activity [36].

Additionally, Helleday et al. found that knocking down MTH1 through siRNA transfection does not inhibit the proliferation of a certain type of cancer cells, while TH588 and TH1579 are more potent in killing cancer cells than siRNA mediated knocking down of MTH1 [29,35]. The discrepancy in data of these two approaches could be due to the development of a compensatory mechanism during the gradual exhaustion of MTH1 protein in the siRNA knocking down approach. While the MTH1 inhibitors in this study allow no time for alternative mechanisms to develop, which argues against the opinion that TH588 and related MTH1 inhibitors have off-targets beyond MTH1.

To further validate the potential of TH287 as a radiotracer for MTH1, full dose range competition binding assays were performed between a well-studied MTH1 inhibitor, (S)-crizotinib, and [^3^H]TH287. Competitive binding assays show that TH287 has a high binding affinity, with a *K*_i_ of 3.04 ± 0.14 nM while (S)-crizotinib shows a *K*_i_ of 153.90 ± 20.48 nM, indicating that its affinity is much lower than TH287. No binding was detected for Raclopride—a nonselective compound (Figure 4). The values of the pseudo-Hill coefficient, *n’*_H_, are 0.88 and 0.70 for TH287 and (S)-crizotinib respectively.

Our competitive assay in U251MG cells shows that the *K*i of TH287 is found to be close to the *IC*_50_ value from pure MTH1 enzymatic assay, and the *n*’_H_ is close to unity, which obeys a single-site binding model. However, the *K*i of (S)-crizotinib is much lower than the *IC*_50_ value from the MTH1 enzymatic assay, and the *n*’_H_ is way off the unity. This suggests that (S)-crizotinib binds to off-targets other than MTH1.

It has been reported that TH287 can be rapidly metabolized in human and mouse liver microsomes through the N-dealkylation of the aminomethyl substituent. The major metabolite is TH586 (*IC*_50_: 77 nM), which has a much higher *IC*_50_ than TH287 (0.8 ± 0.1 nM). It is worth noting that the 11C labeled part is removed and not included in TH586, so TH586 should have minimal impact on applying 11C-TH287 as a radiotracer for PET imaging.

Although the possibility of killing cancer cells through targeting MTH1 is still controversial [30,31,32], a promising yet unexplored application of MTH1 inhibitors is to monitor disease progression that involves oxidative stress since elevated oxidative stress in cells occurs in a large number of chronic diseases including different types of cancers.

Another potential application of this radioligand is to monitor the progression of neurodegenerative diseases such as Alzheimer’s disease (AD) or Parkinson’s disease (PD). In AD, loss of aerobic glycolysis is associated with the presence of neurofibrillary tangles in tau PET studies [37]. Evidence of oxidative stress in diseased brains has been widely reported although direct correlations between upregulated expression of MTH1 and the oxidative status of corresponding brain tissue awaits to be established. Elevated 8-OHG, 8-OHA, and 5,6-diamino-5-formamidopyrimidine have been detected in both nuclear and mitochondrial DNA (mtDNA) extracted from vulnerable brain regions in amnestic mild cognitive impairment, the earliest clinical manifestation of AD, indicating that oxidative stress could be an early event in AD [38]. Thus, monitoring oxidative stress in a noninvasive way though indirectly, through examining MTH1 level in patients’ brains by PET scan, could provide useful information for early intervention.

The work in this study would be essential for further characterizing and developing MTH1 probes for in vitro quantitative autoradiography and in vivo PET to monitor oxidative damage repair. The development of these probes would be highly significant for determining the expression level of MTH1 in both in vitro and in vivo and thus provide a potential marker for therapeutics that target DNA damage and repair.

In conclusion, we have thoroughly evaluated radiolabeled TH287 as a radioligand for MTH1 and compared it with the other unlabeled MTH1 probes: TH086 and (S)-crizotinib. The results of our study indicate that TH287, which possesses a nanomolar affinity for MTH1 is a superior ligand to (S)-crizotinib for measuring MTH1 density and function using in vitro binding assays. TH287 exhibits enrichment in brain tissue and penetrates the blood-brain barrier with a fast clearance rate. The knowledge gained from this study thus lays a solid foundation for further development of radiotracers for in vitro quantitative autoradiography and in vivo PET and will have broad applications to a variety of cancers and other noteworthy diseases (CNS disorders, diabetes, cardiovascular injuries, etc.).

## 4. Materials and Methods

### 4.1. Materials

Chemical reagents and the standard compounds (Raclopride and (S)-crizotinib) were purchased from Sigma-Aldrich (St. Louis, MO, USA) and Tocris (Ellisville, MO, USA). *N*, *N*-Dimethylformamide (DMF), dimethyl sulfoxide (DMSO), or ethanol was used to dissolve the various compounds. Different concentrations were then achieved by diluting stock solutions with assay buffer for the enzymatic assay or RPMI 1640 medium for cell-binding assays. [^3^H]TH287 was customer synthesized by ViTrax (Placentia, CA, USA) via direct catalytic tritium gas exchange, a radiochemical purity of >99% was determined by the high performance liquid chromatography (HPLC). Product identity was confirmed by Mass spectrometry and HPLC co-elution with Authentic Standard (Retention time: 8.39 min; Column: Zorbax SB-Phenyl, 4.6 × 150mm, 3.55 µm; Gradient elution: mobile phase A (0.05% TFA/water) and mobile phase B (acetonitrile). From 10% B to 90% B over 10 min at flow rate 1 mL/min.).Specific Activity was determined to be 31.2 Ci/mmol by Mass Spec. The location of the tritium label has not been determined, however, can be assumed to be located on the aromatic ring.

### 4.2. Synthesis of Precursor and Radiosynthesis of [^11^C]TH287

#### 4.2.1. Synthesis of Precursor

**Scheme 1:**
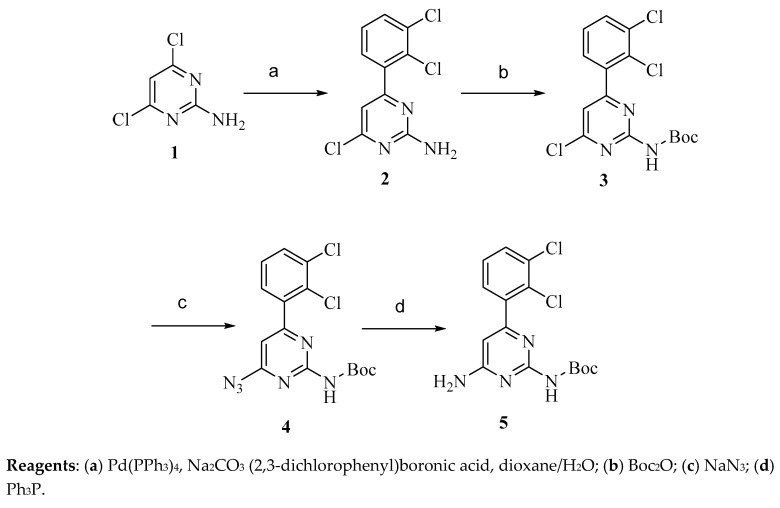
Synthesis of TH287 standards and precursors for radiolabeling with ^11^C.

The synthesis of the C-11 labeling precursor of TH287 (**5**) is shown in Scheme 1. At first, compound **2** was synthesized by coupling dichlorophenyl boronic acid and 4,6-dichloropyrimidin-2-amine (**1**) with Pd(PPh_3_)_4_ as the catalyst. The amine group of **2** was then protected with a Boc group to give compound **3**. The chloro group of compound **3** was substituted with an azide group to afford compound **4**. the azide group of **4** was reduced to an amine group with PPh_3_ to give the precursor **5**.

All chemicals were obtained from standard commercial sources and used without further purification. All reactions were carried out by standard air-free and moisture-free techniques under an inert nitrogen atmosphere with dry solvents unless otherwise stated. Flash column chromatography was conducted using Scientific Adsorbents, Inc. silica gel, 60A, “40 Micron Flash” (32–63 µm). Melting points were determined by using the MEL-TEMP 3.0 apparatus and are uncorrected. Routine ^1^H spectra were recorded at 400 MHz on Agilent Technologies spectrometers. All chemical shifts were reported as a part per million (ppm) downfield from tetramethylsilane (TMS). All coupling constants (*J*) are given in Hertz (Hz). Splitting patterns are typically described as follows: s, singlet; d, doublet; t, triplet; m, multiplet.

**4-Chloro-6-(2,3-dichlorophenyl)pyrimidin-2-amine (2)** A solution of (2,3-dichlorophenyl)boronic acid (640 mg, 3.35 mmol) and 4,6-dichloropyrimidin-2-amine (500 mg, 3.0 mmol) in dioxane (30 mL) and water (10 mL) was added Pd(PPh_3_)_4_ (116 mg, 0.1 mmol) and Na_2_CO_3_ (646 mg, 6.10 mmol). The mixture was heated at 90 °C for 4 h. At room temperature, the reaction mixture was diluted with ethyl acetate (100 mL), washed with water (50 mL × 2) and NaCl solution (75 mL), and dried over Na_2_SO_4_. After evaporation of the solvents, the crude product was purified by column chromatography with CH_2_Cl_2_/EtOAc (5:1) to afford 306 mg (37%) of **2** as a white solid, mp 210.6–211.7 **°**C. ^1^H NMR (400 MHz, CDCl_3_) δ 7.56 (dd, *J* = 8.0 Hz, *J* = 1.2 Hz, 1H), 7.40 (dd, *J* = 7.4 Hz, *J* = 1.2 Hz, 1H), 7.31 (t, *J* = 8.0 Hz, 1H), 6.93 (s, 1H), 5.31 (s, 2H).

***tert*-Butyl (4-chloro-6-(2,3-dichlorophenyl)pyrimidin-2-yl)carbamate (3)** A solution of **2** (275 mg, 1.0 mmol) and di-*tert*-butyl dicarbonate (273 mg, 1.25 mmol) in THF (20 mL) was added *N*,*N*-dimethylpyridin-4-amine (122 mg, 1 mmol). The mixture was stirred at room temperature overnight. After evaporation of the THF, the crude product was purified by column chromatography with CH_2_Cl_2_ to afford 296 mg (79%) of **3** as a colorless oil. ^1^H NMR (400 MHz, CDCl_3_) δ 7.90 (s, 1H), 7.61 (d, *J* = 8.0 Hz, 1H), 7.56 (d, *J* = 7.6 Hz, 1H), 7.37 (t, *J* = 8.0 Hz, 1H), 1.46 (s, 9H).

***tert*-Butyl (4-azido-6-(2,3-dichlorophenyl)pyrimidin-2-yl)carbamate (4)** A solution of **3** (250 mg, 0.67 mmol) and NaN_3_ (130 mg, 2.0 mmol) in DMF (10 mL) was heated at 90 °C for 24 h. At room temperature, the reaction mixture was diluted with ethyl acetate (75 mL), washed with water (50 mL × 2), and NaCl solution (50 mL), and dried over Na_2_SO_4_. After evaporation of the ethyl acetate, the crude product was purified by column chromatography with CH_2_Cl_2_ to afford 216 mg (85%) of **4** as a colorless oil. ^1^H NMR (400 MHz, CDCl_3_) δ 7.54 (d, *J* = 8.4 Hz, 1H), 7.50 (d, *J* = 3.2 Hz, 1H), 7.30 (t, *J* = 7.6 Hz, 1H), 6.78 (s, 1H), 1.55 (s, 9H).

***tert*-Butyl (4-amino-6-(2,3-dichlorophenyl)pyrimidin-2-yl)carbamate (5)** A solution of **4** (150 mg, 0.39 mmol) and Ph_3_P (205 mg, 0.78 mmol) in THF (10 mL) and water (1 mL) was heated to reflux for 4 h to complete the reaction. At room temperature, the reaction mixture was added ethyl acetate (50 mL), and then was washed with water (30 mL × 2) and NaCl solution (30 mL) and dried over Na_2_SO_4_. After evaporation of the ethyl acetate, the crude product was purified by column chromatography with CH_2_Cl_2_/EtOAc (1:1) to afford 116 mg (83%) of **5** as a colorless oil. ^1^H NMR (400 MHz, CDCl_3_) δ 7.65 (s, 1H), 7.50 (d, *J* = 8.4 Hz, 1H), 7.41 (d, *J* = 6.8 Hz, 1H), 7.26 (t, *J* = 8.0 Hz, 1H), 6.36 (s, 1H), 5.39 (s, 2H), 1.51 (s, 9H).

#### 4.2.2. Radiosynthesis of [^11^C]TH287

**Scheme 2:**
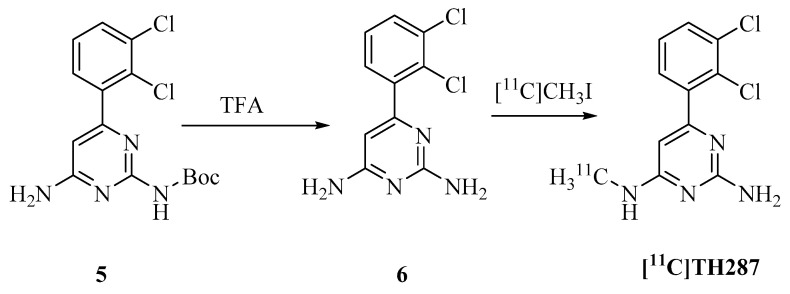
Synthesis of ^11^C labeled MTH1 inhibitors [^11^C]TH287.

The C-11 radiolabeling of the precursor **5** to prepare [^11^C]TH287 is shown in Scheme 2. As previously described [39], [^11^C]CH_3_I was produced from [^11^C]CO_2_ using a GE PETtrace MeI Microlab. [^11^C]CO_2_ was produced at the Cyclotron Facility of Washington University School of Medicine using a JSW BC-16/8 cyclotron by irradiating a gas target of 0.2% O_2_ in N_2_ for 15–30 min with a 40 µA beam of 16 MeV protons. [^11^C]CO_2_ was converted to [^11^C]CH_3_I by the GE PETtrace MeI Microlab using a nickel catalyst [Shimalite-Ni (reduced), Shimadzu, Japan P.N.221-27719] in the presence of H_2_ at 360 °C and followed by a reaction with iodine in the gas phase at 690 °C. [^11^C]CH_3_I was delivered in the gas phase with helium approximately 12 min following the end of the bombardment.

The boc-protected precursor **5** (1.1 mg) was treated with TFA (300 µL) at 80 °C for 10 min, and then TFA was removed under a flow of nitrogen at 80 °C. The residual TFA was removed by the addition of acetonitrile (1 mL) and heating at 80 °C under a flow of nitrogen. This process was repeated two more times to obtain a white residue, which was transferred to the labeling vessel by DMF (2 × 100 µL). [^11^C]MeI was produced at the Washington University in Saint Louis Cyclotron facility. Before the trapping of [^11^C]MeI, NaH (1 mg) in DMF (100 µL) was added to the labeling vessel and the vessel was kept at 0 °C to trap [^11^C]MeI. After trapping of [^11^C]MeI, the reaction was heated at 90 °C for 6 min and then diluted with 1:1 HPLC mobile phase/water for HPLC purification (Phenomenex Luna 250 × 100 mm, 34% MeCN/66%water/0.1%TFA, flow rate: 4 mL/min, UV: 270 nm). [^11^C]TH287 was collected at 15 min in 50 mL of water and extracted by a Waters C18 cartridge. The cartridge was rinsed with 10 mL of water and [^11^C]TH287 was eluted with ethanol and saline to achieve a final dose of 10% ethanol/saline. The radiochemical yield (decay corrected) was 5.9 ± 3.2% (n = 5), and the specific molar activity at the end of the bombardment was 2494 ± 1603 mCi/µmol (n = 5) and radiochemical purity was >99%. At this preliminary stage of our research, we have yet to optimize the labeling. We believe the low yield is due to the slow methylation reaction on the aromatic amine, being limited by the short half-life of carbon-11. The use of more reactive [C-11]methyl triflate may improve the yield under mild conditions.

### 4.3. Cell Culture

U251MG cells (RRID: CVCL_0021) (originally obtained from Dr. Bigner, Duke University, Durham) were grown in RPMI 1640 medium (Invitrogen, Carlsbad, CA, USA) supplemented with 10% fetal bovine serum (Invitrogen, Carlsbad, CA, USA) and 1% penicillin/streptomycin (Invitrogen, Carlsbad, CA, USA). Cells were maintained in a humidified environment of 5% CO_2_ and 95% air at 37 °C. Short tandem repeat (STR) profiling was carried annually from Cell Line Authentication Service from the University of Arizona Genetics Core to ensure quality and integrity.

### 4.4. Knockdown MTH1 through siRNA Transfection

Reverse transfection of MTH1 siRNA was used to knockdown MTH1 expression and cells transfected with control siRNA served as the experimental control. A Lipofectamine RNAiMAX group only was also used as a control group. Briefly, 0.2 µL Lipofectamine RNAiMAX (Invitrogen, CA, USA) mixed with 0.6 µL of 10 µM siRNA in 20 µL of Opti-MEM was added to each well of the 96-well plates. Then, 100 µL of the cell suspension containing 8000 cells was added to each well. The culture medium was changed after overnight incubation at 37 °C. 48 h after the transfection, saturation binding assays were performed on all experimental groups of cells.

### 4.5. MTH1 Binding Assay

#### 4.5.1. Saturation Binding and Scatchard Analysis

U251MG cells (~16, 000 cells per well) were seeded 24 h before the assay. The cells were incubated with [^3^H]TH287 in a total volume of 150 µL of media at 37 °C in 96 well stripwell tissue culture plates (Fisher Scientific, Pittsburgh, PA, USA) for 30 min. The concentrations of the radioligand ranged from 0.5–15 nM. After incubation, each well was washed with 200 µL of ice-cold PBS and transferred to a vial with 2 mL of scintillation fluid. A liquid scintillation system AccuFLEX LSC-800 (Hitachi Aloka Medical, Ltd. Tokyo, Japan) was used to quantify the bound radioactivity. Nonspecific binding was determined from cells treated with 10 µM TH086. Experiments were repeated three times.

The equilibrium dissociation constant (*K*_d_) and the maximum number of binding sites (*B*_max_) were determined by a linear regression analysis of the transformed data using the Scatchard method [40].

Data from saturation radioligand binding studies were transformed to determine the Hill coefficient, *n*_H_, defined as:

(1) $$\log\frac{B_{s}}{B_{\max}-B_{s}}=\log K_{d}+n_{H}\log L$$

where *L* is the concentration of radioligand. *n*_H_, Hill slope, was determined from Hill plot of $\log\frac{B_{s}}{B_{\max}-B_{s}}$ versus logL.

#### 4.5.2. Competitive Binding

U251MG cells (~16,000 cells per well) were seeded the day before the assay and incubated in a total volume of 150 µL of media with [^3^H]TH287 at 37 °C in 96 well plates for 30 min. The final concentration of the radioligand in each assay was 4 nM. Inhibitors (TH287, (S)-crizotinib) concentrations ranging from 0.1 nM to 10 µM were added to acquire the inhibition curves. After the reaction was completed, the cells were washed once with ice-cold PBS and the bound radioactivity was counted and analyzed as described above. Nonspecific binding was determined from cells treated with 10 µM TH086. Raclopride, a well-reported selective antagonist for dopamine D₂ receptors [41,42], was also used to validate the assay. Raclopride shares a similar molecular weight with TH287, but structurally different from TH287 and it has no reported binding affinity to MTH1.

Data from the competitive binding experiments were modeled using nonlinear regression analysis to determine the concentration of inhibitor that inhibits 50% of the radioligand specific binding (*IC*_50_ value). The competition curves were fitted to a single-site binding model using the following equation:

(2) Bs=B0−[(B0∗I)(IC50+I)]

where, *B*_s_ is the amount of the radioligand bound specifically to the cells (i.e., *B*_s_ = *B*_t_ – *B*_ns_, where *B*_t_ is the total bound radioactivity and *B*_ns_ is the nonspecific binding of the radiotracer), *B*_0_ is the amount of the radioligand bound in the absence of the competitive inhibitor, *I* is the concentration of the competitive inhibitor and the *IC*_50_ is the concentration of the competitive inhibitor that blocks 50% of the total specific binding, or 50% sites occupied by the competitor. The values for *B_ns_* and *B*_0_ were constrained using experimentally derived values. Competitive inhibition constants (*K_i_* values) were calculated from the *IC*_50_ values using the Cheng and Prussoff equation [43]:

(3) Ki=IC501+LtKd

where the *K_d_* values of [^3^H]TH287 (1.97 ± 0.18 nM) were derived from U251MG experiments and *L*_t_ is the concentration of radioligand used in each assay.

Data from competitive radioligand binding studies were transformed to determine the pseudo-Hill coefficient, *n’*_H_, defined as:

(4) $$\log\frac{B_{s}}{B_{0}-B_{s}}=n\prime_{H}\log I-n\prime_{H}\log IC_{50}$$

*n’*_H_, which is the negative of the Hill slope, was readily determined from the plot of $\log\frac{B_{s}}{B_{0}-B_{s}}$ versus logI.

### 4.6. Enzyme Activity Assays

The enzymatic hydrolysis of dGTP by purified human recombinant MTH1 to form dGMP and pyrophosphate was applied for screening purposes using a PPiLightTM inorganic pyrophosphate bioluminescent assay—according to published procedures [29]. After shaking and color development, the plates were analyzed in a Victor 3 Microplate reader (PerkinElmer, Waltham, MA, USA) for luminescence. The inhibition assays were conducted using a series of dilutions of standard compounds and new MTH1 inhibitors. The *IC*_50_ values were determined by fitting a dose-response curve to the data points using nonlinear regression.

### 4.7. MTT Assays

MTT assays were used to test cell toxicity of compounds. 2 × 10^3^ U251MG cells were plated into 96-well plates and treated with different concentrations (0.3, 1, 3, 10, 30 µM) of TH287 or (S)-crizotinib at 37 °C for 72 h. After incubation with MTT for 4 h at 37 °C, the culture medium was aspirated and 200 µL DMSO was added to each well to dissolve the formazan crystals, then the absorbance was measured at 540 nm using a Victor 3 Microplate reader (PerkinElmer, Waltham, MA, USA).

### 4.8. MicroPET Imaging of Normal Mice with [^11^C]TH287

The Washington University Animal Studies Committee approved all procedures used for the mouse experiments described in the present study under ACS# 20,170,165 (approved on 9/1/2017). Overall care of the animals was consistent with The Guide for the Care and Use of Laboratory Animals from the National Research Council and the USDA Animal Care Resource Guide. Four male athymic nude mice (~25 mg, 8 weeks old), purchased from Charles River Laboratories (Wilmington, MA, USA), were included for the preliminary microPET imaging evaluation of MTH1 in vivo. The mice were anesthetized with isoflurane and injected with ~400 µCi of [^11^C]TH287 via the tail vein. The imaging sessions were 1 h dynamic scans using the MicroPET^®^ Focus 220 and Inveon scanners (Siemens Medical Solutions USA Molecular Imaging, Knoxville, TN, USA). Acquired list mode data were histogrammed into a 3D set of sinograms and binned to the following time frames: 5 × 60 s, 5 × 2 min, and 9 × 5 min. Sinogram data was then processed using a filter back-projection algorithm with attenuation and scatter corrections. Regions of interest (ROI) were manually drawn on the brain, heart, and kidney of the mice using 0–10 min summarized images as references. The software Acquisition Sinogram Image PROcessing using IDL’s Virtual Machine^TM^ (ASIPro VM^TM^) (Siemens Medical Solutions USA Molecular Imaging, Knoxville, TN, USA) was used to draw ROI and obtain the radioactivity uptake (nCi/c.c.) curve over the time course of the scan, and the uptake data were further normalized by the injected radioactivity dose to get %I.D./cc.

### 4.9. Data and Statistical Analysis

The data and statistical analysis comply with the recommendations on experimental design and analysis in pharmacology [44]. Statistical analyses and figure plotting were performed using KaleidaGraph (Synergy Software, Reading, PA, USA). One-Way ANOVA and Dunnett’s Multiple Comparison test were adopted for significance analysis. In assessing the significance of comparisons a *p*-value of <0.05 was considered statistically significant. All data were reported as mean ± standard deviation if not otherwise noted.

## Abbreviations

MTH1	MutT homolog 1
HCC	Hepatocellular carcinoma
NUDT1	Nudix Hydrolase 1
PET	Positron emission tomography
CT	Computed tomography
MRI	Magnetic resonance imaging
ROS	Reactive oxygen species
IC_50_	The half-maximal inhibitory concentration
